# Encoding of High Frequencies Improves with Maturation of Action Potential Generation in Cultured Neocortical Neurons

**DOI:** 10.3389/fncel.2017.00028

**Published:** 2017-02-14

**Authors:** Evgeny S. Nikitin, Natalia V. Bal, Aleksey Malyshev, Victor N. Ierusalimsky, Yulia Spivak, Pavel M. Balaban, Maxim Volgushev

**Affiliations:** ^1^Institute of Higher Nervous Activity and Neurophysiology, Russian Academy of SciencesMoscow, Russia; ^2^Department of Psychological Sciences, University of ConnecticutStorrs, CT, USA

**Keywords:** neocortical neurons, cultures, slices, development, action potential, encoding, transfer function

## Abstract

The ability of neocortical neurons to detect and encode rapid changes at their inputs is crucial for basic neuronal computations, such as coincidence detection, precise synchronization of activity and spike-timing dependent plasticity. Indeed, populations of cortical neurons can respond to subtle changes of the input very fast, on a millisecond time scale. Theoretical studies and model simulations linked the encoding abilities of neuronal populations to the fast onset dynamics of action potentials (APs). Experimental results support this idea, however mechanisms of fast onset of APs in cortical neurons remain elusive. Studies in neuronal cultures, that are allowing for accurate control over conditions of growth and microenvironment during the development of neurons and provide better access to the spike initiation zone, may help to shed light on mechanisms of AP generation and encoding. Here we characterize properties of AP encoding in neocortical neurons grown for 11–25 days in culture. We show that encoding of high frequencies improves upon culture maturation, which is accompanied by the development of passive electrophysiological properties and AP generation. The onset of APs becomes faster with culture maturation. Statistical analysis using correlations and linear model approaches identified the onset dynamics of APs as a major predictor of age-dependent changes of encoding. Encoding of high frequencies strongly correlated also with the input resistance of neurons. Finally, we show that maturation of encoding properties of neurons in cultures is similar to the maturation of encoding in neurons studied in slices. These results show that maturation of AP generators and encoding is, to a large extent, determined genetically and takes place even without normal micro-environment and activity of the whole brain *in vivo*. This establishes neuronal cultures as a valid experimental model for studying mechanisms of AP generation and encoding, and their maturation.

## Introduction

Cortical neurons can encode rapidly changing stimuli by phase-locking their spiking to high-frequency components of signals (Köndgen et al., 2008; Boucsein et al., 2009; Higgs and Spain, 2009; Tchumatchenko et al., 2011; Broicher et al., 2012; Ilin et al., 2013). Populations of cortical neurons can detect and respond to subtle changes of the input very fast, on a millisecond time scale (Tchumatchenko et al., 2011; Ilin et al., 2013, 2014; Malyshev et al., 2013). Theoretical studies and results of model simulations linked these abilities to the properties of action potential (AP) generators, specifically to the fast onset dynamics of APs (Brunel et al., 2001; Fourcaud-Trocmé et al., 2003; Naundorf et al., 2005; Wei and Wolf, 2011; Huang et al., 2012; Ilin et al., 2013). This idea is supported by experimental results showing that manipulations which slow down the onset of APs disturb the ability of neurons to phase-lock their spiking to high frequencies and decrease the speed of population responses to fast changes of the input (Ilin et al., 2013; for review see Volgushev, 2016).

Despite the established relationship between encoding of high frequencies, response speed and onset dynamics of APs, the mechanisms of fast onset of APs in cortical neurons remain elusive (Naundorf et al., 2006; Yu et al., 2008). APs in neocortical neurons are initiated in the axon initial segment, about 30–50 μm away from the soma (Stuart and Sakmann, 1994; Stuart et al., 1997; Palmer and Stuart, 2006; Fleidervish et al., 2010; Kole and Stuart, 2012; Baranauskas et al., 2013). Poor accessibility of the axon initial segment in slices hinders further progress in understanding intrinsic mechanisms of AP initiation and encoding. Experimental preparations allowing better access to the spike initiation zone might help to address these questions. Neuronal cultures may provide such an experimental model. The use of cultures proved to be a powerful tool to study diverse aspects of axon initial segment functioning, such as trafficking of channel proteins and other molecules, structural plasticity or changes of excitability (e.g., Grubb and Burrone, 2010; Muir and Kittler, 2014; Evans et al., 2015; Albrecht et al., 2016). Moreover, there are important similarities in development of neurons in cultures and *in vivo*, including development of dendritic morphology, synaptogenesis, maturation of synapses and network activity (Li et al., 1998; De Simoni et al., 2003; Harrill et al., 2015; Schneider et al., 2015).

Here we set to characterize encoding properties of cultured neocortical neurons, and to clarify whether cultures represent a valid experimental model for studying mechanisms of neuronal encoding in the brain. We ask: (i) How coding abilities of cultured neurons change with time *in vitro*; (ii) How these changes are related to maturation of AP generation mechanisms; and (iii) How development of encoding in cultured neurons is related to maturation of neuronal encoding in the whole brain?

## Materials and Methods

All experimental procedures of this study are in compliance with the Guide for the Care and Use of Laboratory Animals approved by the Department of Humanitarian Expertise and Bioethics of RAS, and the US National Institutes of Health regulations. Procedures for preparation of slices were approved by the Institutional Animal Care and Use Committee of the University of Connecticut.

### Cell Cultures

Wistar rat pups (P0–P2) were euthanized by decapitation with sharp scissors. The brains were removed, than cortical tissue was dissected and gently cut into pieces with a sharp blade in ice-cold Hank’s balanced salt solution (Gibco). After having it centrifuged, we treated the tissue with TripLE (Gibco) for 5 min at 36°C. TripLE was inactivated by adding ice-cold Neurobasal Medium (Gibco). Cells were dissociated by trituration and washed with Neurobasal medium followed by centrifugation at 2000 rpm for 2 min. Then the tissue was resuspended in Neurobasal Medium (Gibco) with B-27 supplement (Gibco), GlutaMax (Gibco), and cells were plated onto 12 mm glass coverslips coated with poly-D-lysine (Sigma). The initial plating density of cultures was about 1000 cells per square millimeter in a monolayer. Cultures were housed in a CO_2_ incubator prior to electrophysiological experiments (Aseyev et al., 2013).

**Figure 1:**
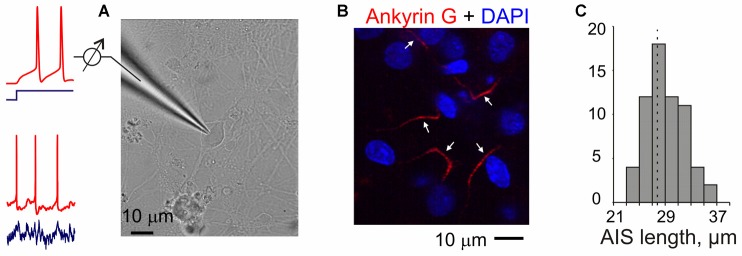
**Experiments in neuron cultures. (A)** Membrane potential responses of cultured neurons to injection of current steps or fluctuating currents were recorded in a whole-cell configuration. **(B)** Example of combined DAPI nuclear staining (blue) and ankyrin-G staining with antibodies labeled with Alexa Fluor Red in the axons of cultured neurons (17 days in culture). Arrows indicate presumable axon initial segments of cultured neurons. Note that a region with small number of clearly isolated axons was selected for illustration and axons of some neurons may be outside of this region; some of the DAPI-staining may also be due to glial cells. **(C)** Distribution of the lengths of axon initial segments measured using ankyrin-G staining in *N* = 63 cultured neurons. Mean length 27.7 ± 0.4 μm.

For electrophysiological recording the glass coverslips with cultured neurons (or brain slices, see below) were placed into a chamber continuously perfused with ASCF containing (in mM): 125 NaCl, 25 NaHCO_3_, 27.5 glucose, 2.5 KCl, 1.25 NaH_2_PO_4_, 2 CaCl_2_ and 1.5 MgCl_2_ (All Sigma Ultra graded), pH 7.4 and preaerated with 95% O_2_, 5% CO_2_. Experiments were performed at near physiological temperature (32–34°C). Recording chamber was mounted on an Olympus BX50W microscope with DIC infrared optics. For recording we selected large cells, and after establishing whole-cell recording configuration, cells with spike shapes similar to those of pyramidal neurons were selected for encoding experiments. Patch pipettes with tip resistances of 5–6 MΩ were filled with a solution containing (in mM): 132 K-Gluconate, 20 KCl, 4 Mg-ATP, 0.3 Na_2_GTP, 10 Na-Phosphocreatine, 10 HEPES, pH 7.25 (all from Sigma, USA). Membrane potential recording and current injection were performed in current clamp mode (Figure 1) using Axoclamp 2B amplifier. Capacitance was compensated for recording APs evoked by depolarizing current steps in the beginning of experiment. These recordings were used to measure parameters of the APs. After that capacitance compensation was reduced to ~60%–70% to avoid occasional overcompensation during recording responses to injection of fluctuating current. Series resistance was not compensated. Recordings were filtered at 10 kHz and digitized at 20 kHz using ADC Digidata 1440 (Axon Instruments, CA, USA) and pCLAMP software (Molecular Devices).

### Immunohistochemistry and Confocal Imaging

Cell cultures were fixed in 2% paraformaldehyde in phosphate-buffered saline for 10 min at room temperature. Prior to the incubation with primary antibodies, the cultures were washed in the blocking solution (0.5% Triton X-100, 0.01% sodium azide, 5% normal goat serum, and 1% BSA in PBS, all from Sigma) for 2 h. Staining was performed in the blocking solution at 4°C in two stages: primary antibodies overnight with washing in for 1 h; secondary antibodies for 12 h with washing in for 1 h. Primary mouse monoclonal anti-ankyrin G antibodies (Calbiochem) were used at 1:500 dilution. Secondary goat anti-mouse Alexa-546-conjugated IgG antibodies (Invitrogen) were used at 1:100 dilution. The sections were embedded in SlowFade Gold antifade reagent with DAPI (Invitrogen). Confocal imaging was performed using Axiovision software. Stacks of images were acquired at 0.6–1 μm steps with the LSM 5 Live confocal scanning microscope and 63×/1.4 N.A. oil objective (Zeiss, Germany) to produce a detailed 3D-reconstruction.

### Experiments in Slices

Procedures for preparation of slices were approved by the Institutional Animal Care and Use Committee of the University of Connecticut. Details of slice preparation are similar to those used in previous studies (Volgushev et al., 2000; Lee et al., 2012; Ilin et al., 2013). Slices were prepared from male Wistar rats (two age groups, P9–P13 and P17–P25) obtained from Charles-River or Harlan. Rats were anesthetized with isoflurane, decapitated, and the brain quickly removed and placed into an ice-cold oxygenated artificial cerebrospinal fluid solution (ACSF), containing, in mM: 125 NaCl, 25 NaHCO_3_, 25 glucose, 3 KCl, 1.25 NaH_2_PO_4_, 2 CaCl_2_, 1 MgCl_2_, bubbled with 95% O_2_/5% CO_2_, pH 7.4. Coronal slices (350 μm thickness) containing the visual cortex were prepared from the right hemisphere. After at least 1 h recovery at room temperature, individual slices were transferred to a recording chamber mounted on an Olympus BX-50WI microscope equipped with IR-DIC optics.

Whole-cell recordings using patch electrodes were made at 28–32°C. Layer 2/3 pyramidal neurons in the visual cortex were selected under visual control using infrared videomicroscopy. The patch electrodes were filled with K-gluconate based solution (in mM: 130 K-Gluconate, 20 KCl, 4 Mg-ATP, 0.3 Na_2_-GTP, 10 Na-Phosphocreatine, 10 HEPES) and had a resistance of 4−6 MΩ. Recordings were made using Axoclamp-2A (Axon Instruments, CA, USA) or Dagan BVC-700A (Dagan Corporation, MN, USA) amplifier. Similar to recordings from cultured neurons, capacitance was compensated for recording APs evoked by depolarizing current steps, and capacitance compensation was reduced to ~60–70% after that, for recording responses to injection of fluctuating current. Series resistance was not compensated. After amplification and low-pass filtering at 10 kHz, data were digitized at 20 kHz and fed into a computer (Digidata 1440A interface and pCLAMP software, Molecular Devices).

### Current Injection and Calculation of Transfer Function

To calculate the frequency transfer function of individual neurons, we injected fluctuating current that mimics the effect produced in the soma by numerous balanced excitatory and inhibitory synaptic inputs (Destexhe et al., 2003). Fluctuating current for injection was synthetized as a summed activity of large population of model presynaptic excitatory (*N* = 512) and inhibitory (*N* = 512) neurons, each firing at mean rate of 5 Hz (Ilin et al., 2014). Individual excitatory and inhibitory postsynaptic currents were generated as a difference of two exponentials with a rise time of 0.5 ms and decay time of 5 ms. Because of the same number of excitatory and inhibitory neurons and same amplitudes (but for the sign) of excitatory and inhibitory currents the resulting fluctuating current was balanced. Injected current was scaled to produce membrane potential fluctuations of 15–20 mV amplitude, similar to membrane potential fluctuations recorded in neocortical neurons *in vivo* (Destexhe et al., 2003; Volgushev et al., 2003). Current injections lasted 46 s, and were separated by a recovery period of 60–100 s. We used 20 different realizations of 46 s-episodes of fluctuating current for injection in neurons in this study.

In the recorded responses to injected current we calculated timing of each AP as a positive-slope crossing of zero potential. Using spike timings we calculated normalized spike triggered average of the injected current and power spectrum of the spike-triggered average (STA) using Fast Fourier Transformation (FFT). Next, normalized autocorrelation function of the injected current and its FFT were calculated. Frequency transfer function of a neuron was then calculated as the ratio of the FFT transfers of the STA and the autocorrelation function of the input current. Significance of the transfer function was calculated as following. The same procedure of calculation of transfer function as above was repeated 500 times, but with shuffled spike times. We then calculated 95th percentile of these “shuffled” transfer functions and used it as a significance margin, above which the transfer function was considered significant. Further details of this method are provided in Ilin et al. (2013). In this prior work we have also demonstrated that transfer function calculated using this noise-injection method is similar to the transfer function calculated using a sine-wave immersed in noise paradigm (Ilin et al., 2013).

### Database

In the final analysis we included 29 neurons recorded in cultures. Most cultured neurons displayed rare spontaneous spiking, and to avoid contamination of STA by spontaneous spikes we only used for analysis neurons with the spontaneous firing rate lower than one spike per minute. Other criteria included stability of the membrane potential, input resistance and responses to injection of fluctuation current. In preliminary experiments we tested if addition of blockers of synaptic transmission changes the transfer function. Because no significant differences between transfer functions measured with or without blockers were found, we pooled results together. For comparison we used data for 25 neurons from slice experiments, some of which were used in a prior study (Ilin et al., 2013).

### Statistical Analysis

Correlations (Spearman-Rho) and their significance were calculated using IBM SPSS Statistics package (PASW Statistics version 18.0.0). Analysis using linear models was done in R (version 3.2.3 (2015-12-10), The R Foundation for Statistical Computing), functions *regsubsets* and *lm*.

## Results

### Action Potential Encoding of High Frequencies Improves with Maturation of Neurons in Culture

To verify that neurons in our cultures developed normal axon initial segments we used anti-ankyrin-G staining. Ankyrin-G is selectively expressed in the axon initial segment of neurons, and is an established label for the axon initial segments (Lorincz and Nusser, 2008; Kole and Stuart, 2012). Staining neurons in 17-day old cultures with anti-ankyrin-G antibodies revealed a characteristic pattern of linear, 20–35 μm long (mean 27.7 ± 0.4 μm, *N* = 63) smooth stripes near the soma, but no staining in the soma, dendrites or dendritic spines (Figures 1B,C). This pattern of staining is similar to that reported in other studies of neurons from brain tissue (Lorincz and Nusser, 2008; Kole and Stuart, 2012; Gutzmann et al., 2014) and in cultures (Grubb and Burrone, 2010; Wefelmeyer et al., 2015), indicating no abnormalities in the development of axon initial segments in our cultures.

**Figure 2:**
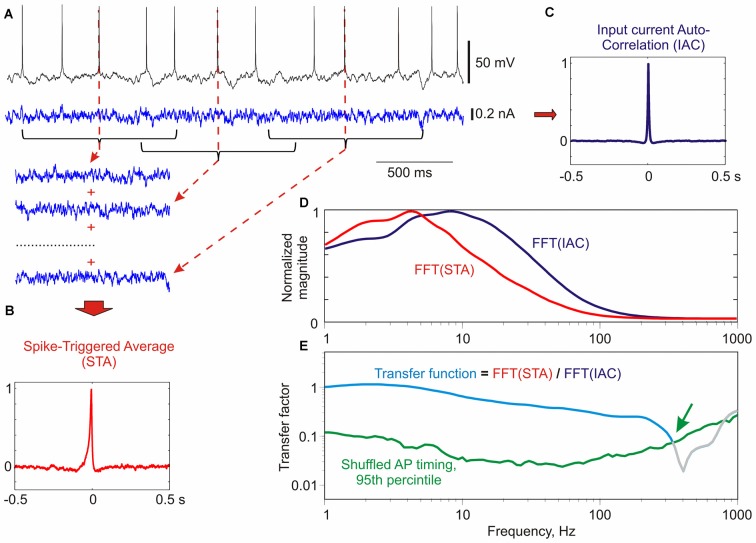
**Calculation of frequency transfer function of a culture neuron using noise injection. (A)** Response of a neuron in culture to injection of fluctuating current. For calculation of spike-triggered average (STA) 1 s portions of the injected current centered at each spike were used. Three such segments are shown for clarity. **(B)** Normalized STA of the injected current. **(C)** Normalized auto-correlation function of the injected current (IAC). **(D)** Normalized Fast Fourier Transformation (FFT) transforms of the spike-triggered average FFT(STA), and of the input auto-correlation FFT(IAC). **(E)** Transfer function (cyan) calculated as the ratio of the normalized FFT transforms of the STA and the input auto-correlation. Green line shows 95th percentile of “shuffled” transfer functions (*N* = 500), calculated as above, but using shuffled instead of real timings of spikes. Values above the 95th percentile of shuffled transfer function are considered significant. The transfer function was cut at the intersection with the 95th percentile (green arrow). Method of calculation of transfer function is based on Higgs and Spain (2009); significance estimation as in Ilin et al. (2013).

We measured encoding properties of cultured neurons using frequency transfer function. Frequency transfer function provides a comprehensive characterization of the ability of neurons to encode periodic signals of different frequencies by phase-locking the generation of APs to a specific phase of the sine wave (Köndgen et al., 2008; Higgs and Spain, 2009; Ilin et al., 2013). The transfer function was calculated as following (Figure 2; for details see Ilin et al., 2013). We measured membrane potential response of neurons to injection of fluctuating noise through the recording electrode, and determined the timing of APs generated by the neuron. Next, we calculated the spike triggered average (Figure 2B) and autocorrelation function (Figure 2C) of the injected current. Transfer function was then calculated as the ratio of the FFT transforms of the STA and the autocorrelation function of the input current (Figures 2D,E). Calculated transfer function shows that the cultured neuron in this example was able to phase-lock generation of APs to signal frequencies of up to ~300 Hz (Figure 2E, green arrow).

**Figure 3:**
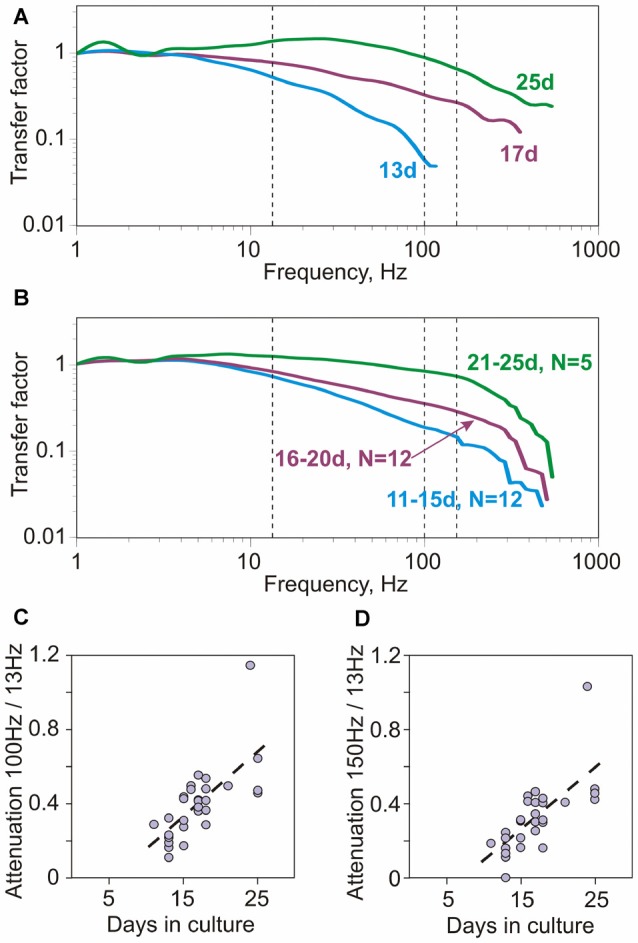
**Encoding of high frequencies in cultured neurons improves with age (days in culture). (A)** Example transfer functions of three neurons, cultured for 13, 17 and 25 days. Vertical dashed lines show 13 Hz, 100 Hz and 150 Hz. Averaged firing rate for the three neurons was 4.18 Hz; 5.08 Hz and 4.6 Hz, respectively. **(B)** Group-averages of transfer functions of neurons cultured for 11–15 days (*N* = 12), 16–20 days (*N* = 12) and 21–25 days (*N* = 5). **(C,D)** Correlation between the attenuation of encoding of high frequencies and age (days in culture). Attenuation of encoding of high frequencies was quantified using the ratio of transfer factor at 100 Hz over the transfer factor at 13 Hz (**C**, 100 Hz/13 Hz) or the ratio of factors at 150 Hz over 13 Hz (**D**, 150 Hz/13 Hz). Both measures were strongly correlated with age (Spearman-Rho 0.72, *p* < 0.001 and 0.73, *p* < 0.001; *N* = 29).

How does spike encoding change with development of neurons in culture? Figure 3A shows transfer functions of three neurons recorded after growing in culture for 13, 17 or 25 days. The ability of neurons to phase-lock their spiking to high frequencies systematically increases with age. While the neuron from 13-day old culture was not able to phase-lock spiking to signal frequencies above 100 Hz (Figure 3A, cyan), in neurons from older cultures the range of encoded frequencies expanded and transfer factors for higher frequency range increased. In the neuron from 25 day-old culture the range of encoded frequencies reached 400–500 Hz (Figure 3A, green). The improvement of encoding of high frequencies with culture age is further illustrated by the group-averaged transfer functions of neurons grown in culture for 11–15 days, 16–20 days and 21–25 days (Figure 3B). To quantify the ability of neurons to encode high frequencies we calculated the ratio of the transfer factors at 100 Hz to 13 Hz, and at 150 Hz to 13 Hz. Both measures were strongly correlated with the age of culture (Spearman-Rho 0.72, *p* < 0.001 and 0.73, *p* < 0.001; *N* = 29; Figures 3C,D).

Thus, encoding of high frequencies correlated with the age of culture, and improved significantly over the period from 11 to 25 days.

### Maturation of Membrane Properties and Action Potential (AP) Generation in Neurons in Culture

**Figure 4:**
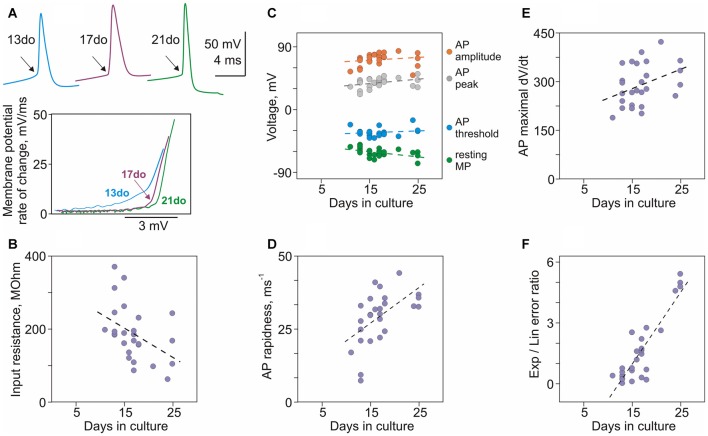
**Maturation of membrane properties and action potential (AP) generation in cultured neurons. (A)** APs from three neurons cultured for 13, 17 and 21 days, and their onsets in phase plots. In the phase plots, APs were shifted along the X-scale (voltage) to facilitate comparison of their onset dynamics. **(B)** Decrease of input resistance with age (*r* = −0.49, *p* = 0.006, *N* = 29). **(C)** Correlation of resting membrane potential (*r* = −0.50, *p* = 0.006), and AP threshold (*r* = 0.003, *p* = 0.99), peak (*r* = 0.36, *p* = 0.053) and amplitude (*r* = 0.42, *p* = 0.024) with age of cultured neurons. **(D)** AP rapidness increases with age (*r* = 0.65, *p* < 0.001). **(E)** Maximal rate of rise of APs increases with age (*r* = 0.46, *p* = 0.013). **(F)** The ratio of errors of the exponential and the linear fits of AP onset increases with age (*r* = 0.63, *p* < 0.001).

A number of basic electrophysiological properties and characteristics of APs changed dramatically in neurons between 11 and 25 days in culture, and were significantly correlated with the culture age (Figures 4A–F). Resting membrane potential, input resistance and membrane time constant were negatively correlated with age: neurons became more hyperpolarized (*r* = −0.50, *p* = 0.006; *N* = 29; Figure 4C, green circles), their input resistance decreased (*r* = −0.49, *p* = 0.006; Figure 4B), and time constant had a tendency to become shorter (*r* = −0.27, *p* = 0.15). Most prominent changes in the generation of APs were their more rapid onset and faster rising front. A characteristic kink at the onset of an AP in conventional voltage-over-time plots became more evident in spikes generated by older neurons (Figure 4A, top traces, arrows). Faster onset in older neurons was especially clear in the phase-plots of initial portions of spikes (Figure 4A). For quantification of onset dynamics of spikes we used two measures: rapidness (slope of the phase plot at 20 mV/ms; Naundorf et al., 2006) and the ratio of errors of the exponential and linear fits of AP onset in the phase plot (Volgushev et al., 2008). Both measures strongly correlated with culture age (rapidness: *r* = 0.65, *p* < 0.001, Figure 4D; ratio of exponential-to-linear fit errors: *r* = 0.63, *p* < 0.001, Figure 4F). Increase of the speed of AP rise with age is evidenced by strong positive correlation between culture age and maximal rate of voltage increase (maximal dV/dt, Figure 4E, *r* = 0.46, *p* = 0.013).

Other characteristics of APs either weakly correlated or did not change with age of cultures. Weak correlations with age were found for AP amplitude (*r* = 0.42, *p* = 0.024) and peak (*r* = 0.36, *p* = 0.053; Figure 4C). No correlation with age was found for AP threshold (*r* = 0.003, *p* = 0.99; Figure 4C) and width at half-amplitude (*r* = −0.072, *p* = 0.71; not shown).

Thus, development of neurons between 11 and 25 days in culture is associated with increasingly hyperpolarized membrane potential, decreasing input resistance and faster onset and upstroke of APs.

### Which Factors Determine Encoding Properties of Neurons in Culture?

We used several statistical approaches to reveal which factors determine maturation of encoding properties of neurons in culture, specifically the improvement of encoding of high frequencies by neurons from older cultures.

**Figure 5:**
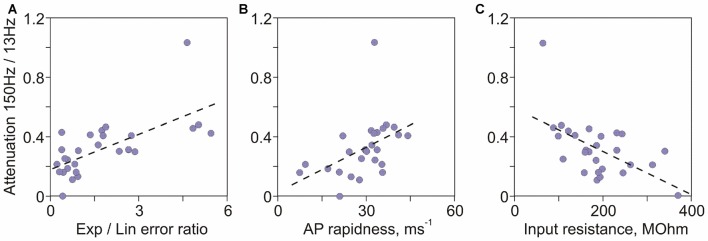
**Factors determining encoding of high frequencies in cultured neurons. (A)** Correlation between the attenuation of high frequency encoding (ratio of transfer factors at 150 Hz over 13 Hz) and the ratio of errors of exponential to linear fits of AP onset (*r* = 0.65, *p* < 0.001). **(B)** Correlation between the attenuation of 150 Hz/13 Hz encoding and AP onset rapidness (*r* = 0.64, *p* < 0.001). **(C)** Correlation between the attenuation of 150 Hz/13 Hz encoding and input resistance (*r* = −0.53, *p* = 0.003).

In the first approach we analyzed correlations between a quantitative measure of high frequency encoding, the ratio of transfer factors for 150 Hz and 13 Hz on the one hand, and age of culture and electrophysiological characteristics of neurons on the other. The ratio of 150 Hz/13 Hz transfer factors correlated most strongly with age of cultures (Spearman-Rho = 0.73, *R*^2^ = 0.54, *p* < 0.001; Figure 3D), indicating that age of neurons in culture accounts for >50% of the variance of their encoding abilities. The strongest predictive power of age is not surprising, because age is the common factor that governs changes of all electrophysiological properties of neurons. Other significant factors, in the order of *R*^2^ values were: ratio of errors of exponential to linear fits of the AP onset (*R*^2^ = 0.42, *p* < 0.001; Figure 5A), AP rapidness (*R*^2^ = 0.41, *p* < 0.001; Figure 5B), input resistance (*R*^2^ = 0.28, *p* = 0.003; Figure 5C), followed by resting membrane potential (*R*^2^ = 0.2, *p* = 0.015) and AP amplitude (*R*^2^ = 0.15, *p* = 0.038). Ability to encode high frequencies did not correlate with other measured characteristics of APs: maximal rate of rise (*R*^2^ = 0.09, *p* = 0.12), peak (*R*^2^ = 0.08, *p* = 0.13), threshold (*R*^2^ = 0.001, *p* = 0.89) and width at half amplitude (*R*^2^ = 0.0002, *p* = 0.94). Thus, among measured electrophysiological characteristics of neurons, two measures of the onset of AP—the ratio of errors of exponential to linear fits and onset rapidness—were strongest predictors of abilities of neurons to encode high frequencies. The next strongest factor was input resistance.

In the second approach we used linear model, in which the ratio of 150 Hz/13 Hz transfer factors was considered a response, and all other measured electrophysiological characteristics of neurons (resting membrane potential, input resistance, AP threshold, rapidness, maximal rate of rise, peak, amplitude, width at half height and the ratio of errors of exponential to linear fits) were considered predictors. For subsets of each size (1–8) the optimal combination of these predictors that minimized the residual standard error was determined (function *regsubsets* in R version 3.2.3 (2015-12-10), The R Foundation for Statistical Computing). Optimal subsets of all sizes (from 1 up to 8) contained the quantitative measure of AP onset dynamics, the ratio of exponential-to-linear fit errors, supporting the conclusion that onset dynamics was the strongest predictor of the neurons’ ability to encode high frequencies.

Finally, in the linear model which included all nine of the above predictors of encoding of high frequencies (*F*_DF__(9,18)_ = 3.256, *p* = 0.016) the strongest predictor was again the ratio of exponential to linear fit errors (*t* = 2.592, *p* = 0.018; all other factors *p* > 0.1; function *lm* in R version 3.2.3).

Thus, results of all three statistical approaches identified onset dynamics of APs as the major factor determining abilities of neurons to encode high frequency signals.

### Similarity of Age-Dependent Improvement of Encoding in Neurons in Culture and in Acute Slices

**Figure 6:**
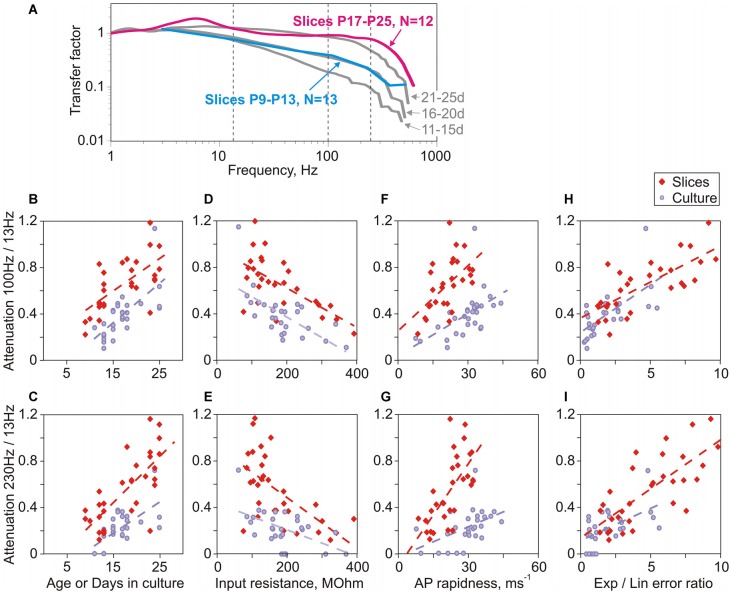
**Parallels in the age-dependent improvement of encoding of high frequencies in neurons in slices and in cultures. (A)** Averaged transfer functions for two groups of neurons in slices prepared from 9 to 13 day old rats (cyan, *N* = 13) and 17–25 day old animals (magenta, *N* = 12). For comparison, transfer functions for three age groups of cultured neurons are shown (gray; 11–15 day; 16–20 day and 21–25 day; data from Figure 3B). **(B,C)** Correlation between attenuation of encoding of high frequencies (**B**: 100 Hz/13 Hz; **C**: 230 Hz/13 Hz) and age for slice neurons (red diamond symbols, **B**: *r* = 0.35, *p* = 0.056; **C**: *r* = 0.72, *p* < 0.001) or days in culture for cultured neurons (lilac circles, **B**: *r* = 0.72, *p* < 0.001; **C**: *r* = 0.63, *p* < 0.001). **(D,E)** Correlation between attenuation of encoding of high frequencies (**D**: 100 Hz/13 Hz; **E**: 230 Hz/13 Hz) and input resistance for neurons in slices (red diamonds, **D**: *r* = −0.41, *p* = 0.022; **E**: *r* = −0.62, *p* < 0.001) and in cultures (lilac circles, **D**: *r* = −0.71, *p* < 0.001; **E**: *r* = −0.45, *p* = 0.013). **(F,G)** Correlation between attenuation of encoding of high frequencies (**F**: 100 Hz/13 Hz; **G**: 230 Hz/13 Hz) and AP onset rapidness for neurons in slices (red diamonds, **F**: *r* = 0.28, *p* = 0.12; **G**: *r* = 0.72, *p* < 0.001) and in cultures (lilac circles, **F**: *r* = 0.71, *p* < 0.001; **G**: *r* = 0.54, *p* = 0.002). **(H,I)** Correlation between attenuation of encoding of high frequencies (**H**: 100 Hz/13 Hz; **I**: 230 Hz/13 Hz) and the ratio of errors of exponential to linear fits for neurons in slices (red diamonds, **H**: *r* = 0.55, *p* = 0.001; **I**: *r* = 0.75, *p* < 0.001) and in cultures (lilac circles, **H**: *r* = 0.69, *p* < 0.001; **I**: *r* = 0.53, *p* = 0.003).

In prior work with neurons in slices we found that encoding of high frequencies improves with age (Ilin et al., 2013). Here we ask, how does maturation of encoding properties of neurons in cultures compares to that during normal development in the whole brain *in vivo*, assessed with recordings from slices prepared from animals of different ages. We found several marked similarities between maturation of encoding in cultures and *in vivo*. First, comparison of transfer functions of neurons in slices prepared from animals of two age groups, P9–P13 and P17–P25, revealed clear improvement of encoding of high frequencies with age (Figure 6A). Second, there was a strong correlation between quantitative measures of encoding of high frequencies (ratio of transfer factors at 100 Hz/13 Hz and at 230 Hz/13 Hz) on the one hand, and age on the other (Figure 6B, cultures: *r* = 0.72, *p* < 0.001, slices: *r* = 0.35, *p* = 0.056 and Figure 6C, cultures: *r* = 0.63, *p* < 0.001, slices: *r* = 0.72, *p* < 0.001). Improvement of encoding with age was also strongly correlated with the decrease of the input resistance (Figure 6D, cultures: *r* = −0.71, *p* < 0.001, slices: *r* = −0.41, *p* = 0.022; and Figure 6E, cultures: *r* = −0.45, *p* = 0.013; slices: *r* = −0.62, *p* < 0.001). Third, in neurons both from slices and cultures, encoding of high frequencies correlated with measures of AP onset, the ratio of errors of exponential and linear fits of AP onset (Figures 6H,I, cultures: *r* = 0.69, *p* < 0.001 and *r* = 0.53, *p* = 0.003; slices: *r* = 0.55, *p* = 0.001 and *r* = 0.75, *p* < 0.001) and the AP rapidness (Figures 6F,G, cultures: *r* = 0.71, *p* < 0.001 and *r* = 0.54, *p* = 0.002; slices: *r* = 0.28, *p* = 0.12 and *r* = 0.72, *p* < 0.001).

Aside from these clear similarities, one notable difference was a better encoding of high frequencies by neurons from slices as compared to matched-age neurons from cultures. This is evidenced by comparison of transfer functions of group-averages (Figure 6A). Further, in the plots of encoding against age, input resistance and AP rapidness data-points representing neurons from slices and from cultures form separable clouds, with data-points for slices shifted upwards relative to those from cultures (Figures 6B–G, red vs. lilac symbols). In contrast, in the plots of encoding against the ratio of errors of exponential and linear fits of AP onset all data points form one cloud, with similar regression lines for data from slices and from cultures. Neurons from slices had faster onset of APs as evidenced by higher values of the error ratio, and encoded high frequencies better than neurons from cultures (Figures 6H,I). Thus the reason for better encoding in neurons from slices could be faster onset of their APs. We interpret these results as an indication that, among the parameters considered above, the AP onset quantified with the error ratio is the strongest predictor of encoding of high frequencies.

To summarize, neurons developed in cultures or in the whole brain express clear similarities of the maturation of AP onset dynamics and encoding. Notably, this similarity occurs in spite of dramatically different environment to which neurons are exposed during the development.

## Discussion

Results of the present study demonstrate that, in cultured neocortical neurons: (i) encoding of high frequencies improves with maturation of the cultures, in parallel with development of passive electrophysiological properties and AP generation; (ii) the major predictor of encoding is the onset dynamics of APs; and (iii) maturation of encoding properties is similar to developmental changes of encoding in neurons studied in slices.

### Electrophysiological Properties and Action Potential Generation in Neurons in Cultures

Passive membrane properties and generation of APs change between 11 and 25 days in culture. Neurons become more hyperpolarized, their input resistance decreases, and membrane time constant becomes shorter. These changes suggest an increase of potassium conductance at rest. An additional factor contributing to a decrease of input resistance might be an increase of size and membrane area of neurons, as reported for both organotypic and dissociated cultures (De Simoni et al., 2003; Harrill et al., 2015). At the same time, threshold for AP generation remained stable over that period. This combination of changes implies a decrease of excitability of neurons: the distance from a more hyperpolarized membrane potential to the unchanged threshold potential increases, and because of the decreased input resistance more input (synaptic) current would be needed to depolarize the membrane to the threshold.

Several lines of evidence indicate changes of the spike generation mechanisms in cultured neurons over the studied period. First, because the rate of rise of an AP is proportional to sodium current density, the increase of the maximal rate of rise implies an increase of the density of sodium current (and sodium channels). Faster onset of APs, documented by the increase of the rapidness and the ratio of errors of exponential and linear fits of AP onset can be due to a shift of the initiation zone of APs down the axon initial segment, leading to the “invasion current” from the generation site recorded at the beginning of the AP in the soma (McCormick et al., 2007; Yu et al., 2008). Both the increase of the maximal rate of rise and faster onset of APs can be also suggestive of a faster gating kinetics of sodium channels in the axon initial segment (Baranauskas and Martina, 2006; Naundorf et al., 2006; Schmidt-Hieber and Bischofberger, 2010). Identifying how each of these three factors change during development of neurons in cultures, and how their interaction leads to the observed changes of spike generation requires further investigation.

### Which Factors Mediate the Enhancement of Encoding with Age of Neurons in Culture?

Clear improvement of encoding of high frequencies in cultured neurons and its strong correlation with age over the studied period (from day 11 to day 25 in culture) provide an opportunity to analyze which factors determine age-dependent changes of encoding. Theoretical studies predicted that major determinant of encoding properties of neuronal populations is the onset dynamics of APs (Fourcaud-Trocmé et al., 2003; Naundorf et al., 2005; Wei and Wolf, 2011; Huang et al., 2012). Prior research provided several lines of evidence in support of this prediction. Experiments with neurons in slices showed that encoding of high frequencies correlated with the onset rapidness of APs, and manipulations that slowed-down the onset of spikes impaired the ability of neurons to encode high frequencies (Ilin et al., 2013). Mature neurons with fast-onset spikes encoded high frequencies better than neurons in slices from younger animals with immature spike generators and slow-onset spikes (Ilin et al., 2013). Results of the present study provide further support to this idea. Statistical analysis using correlations and linear model approaches identified the quantitative measure of AP onset dynamic—the ratio of errors of exponential and linear fits of spike onset, as major predictor of age-dependent changes of encoding. Encoding of high frequencies was also strongly correlated with a second measure of onset dynamics, AP rapidness.

Recent modeling work predicted that dendritic load is one further factor that determines encoding: with increasing dendritic load, model neurons were able to encode progressively higher frequencies (Eyal et al., 2014). Experimental results showing better encoding of high frequencies by large L5 pyramids than by small L2/3 pyramids support this conjecture (Volgushev, 2016). Present results lend further support to this notion: improvement of encoding of high frequencies with age was correlated with the decrease of input resistance, which might be at least partially related to the larger dendritic tree of neurons from older cultures (De Simoni et al., 2003; Harrill et al., 2015) and thus increased dendritic load.

Other electrophysiological properties of neurons measured in this study expressed only a weak or no correlation with the ability to encode high frequencies (resting potential *r* = −0.45, *p* = 0.15; AP amplitude *r* = 0.39, *p* = 0.038; no correlation for membrane time constant, AP threshold, maximal rate of rise, peak and width). Notably, linear model approach did not identify AP amplitude and rate of rise as significant predictors of encoding abilities. Because AP rate of rise is proportional to the density of sodium current (and channels), this result indicates that density of sodium channels as such is not a significant predictor of encoding. This is consistent with modeling results which demonstrated that increasing sodium current density in model neurons did not lead to significant improvement of encoding (Huang et al., 2012; Ilin et al., 2013).

Changes of excitability, AP generation and encoding abilities of neurons might alter the ability of neuronal networks in cultures to generate precisely patterned activity and thus contribute to the reported changes of activity patterns with age of cultures (Maeda et al., 1995; Harrill et al., 2015; Schneider et al., 2015). Such age-dependent changes might also have implications for recently suggested construction of large scale logical devices exploiting geometrical design of neural cultures *in vitro* (Feinerman et al., 2008; Kato-Negishi et al., 2013). The observed dependence of the AP generation and encoding on culture age may negatively affect the reproducibility of computations in such devices.

### Outlook: Cultured Neurons as an Experimental Model for Studying Action Potential Encoding

Results of the present study revealed clear similarity between the development of encoding properties in neurons in cultures and maturation of encoding properties *in vivo*, as accessed in experiments in acute slices prepared from animals of different ages. In both cases ability of neurons to encode high frequencies was significantly correlated with age, improving with maturation of neurons. Further, encoding of high frequencies correlated with the onset dynamics of APs, and was enhanced in neurons with faster AP onset dynamics. Quantitative measures of AP onset dynamics were the strongest predictors of the encoding properties of neurons. These similarities were present despite strikingly different conditions in which neurons developed: cellular micro-environment, interactions with other neurons and activity in cultures is dramatically simplified compared to the whole brain *in vivo*. Parallels in maturation of AP generation and encoding in neurons in cultures and *in vivo* indicate that these processes are to a large extent determined by genetic mechanisms governing development of nerve cells. Support to this notion comes from studies which demonstrated further similarities between development of neurons in cultures and *in vivo*, including dendritic growth, synaptogenesis, and maturation of synaptic and network activity (Li et al., 1998; De Simoni et al., 2003; Harrill et al., 2015; Schneider et al., 2015). This makes cultures a suitable and valuable experimental model to study the development of AP generation and encoding, and allows to exploit advantages of cultures over experiments in slices. These advantages include accurate control over conditions of growth and microenvironment throughout the development, a possibility to precisely modify these conditions (e.g., Grubb and Burrone, 2010), and enhanced access to the axon initial segment (e.g., Albrecht et al., 2016; Muir and Kittler, 2014). Further, experiments in cultures expand the range of genetic tools applicable to study AP generation and encoding, e.g., allowing the use of mutations that affect sodium channels and have severe physiological consequences (e.g., Scn8a mutation, in which Nav1.6 do not replace Nav1.2, leading to severe dysfunction and lethality around PD21; Burgess et al., 1995; Meisler et al., 2001).

Interestingly, results from cultured neurons had a better correspondence with the predictions of computer models. Similar to computer models (Huang et al., 2012; Ilin et al., 2013), encoding in cultured neurons correlated with AP onset and input resistance, but not with other parameters of AP, such as rate of rise or amplitude. Prior work in slices found correlation of encoding with AP rate of rise and amplitude (Ilin et al., 2013). Possible reasons for this discrepancy may include influence of natural environment and natural activity of the whole brain on the development of neurons. Lower number of synapses and less elaborated dendritic tree of neurons in cultures as compared to slices (De Simoni et al., 2003; Harrill et al., 2015) could be one of the reasons why models with simple morphology (Ilin et al., 2013) better captured properties of cultured neurons. One further reason could be that during normal development in the whole brain *in vivo* factors additional to genetically-determined program executed in cultures were activated, leading to a faster onset of APs and better encoding of high frequencies as our results show, but also to deviations of real neuron behavior from that of a simple model. Among these factors could be up-regulation and redistribution of potassium channels and increased density of sodium channels in the axon initial segment, as well as a shift of the AP generation zone. Indeed, these factors change during normal development, and sensory deprivation disrupts refinement of the structure and location of the axon initial segment (Gutzmann et al., 2014; Kuba et al., 2014; Adachi et al., 2015; Yamada and Kuba, 2016), pointing at the involvement of activity-dependent mechanisms in final shaping of spike generation zone (Grubb and Burrone, 2010; Evans et al., 2015; Wefelmeyer et al., 2015). A further reason for enhanced encoding of high frequencies in neurons in slices could be higher dendritic load (Eyal et al., 2014), due to both, larger dendritic trees and higher level of spontaneous activity in neurons in acute slices as compared to cultures (De Simoni et al., 2003). Experiments with neuronal cultures, in which conditions of growth can be precisely controlled and modified, provide a unique opportunity to disentangle the contribution of genetically-determined program and activity-dependent mechanisms to the development of AP generators and encoding. Understanding these factors might provide further insights into mechanisms of AP generation and encoding in cortical neurons and their maturation.

## Author Contributions

ESN, AM, PMB and MV: designed the study; ESN, NVB, AM, VNI and YS: performed experiments; ESN, AM, VNI and MV: processed results; ESN and MV: wrote the manuscript. All authors have read and approved the submitted version of the manuscript.

## Conflict of Interest Statement

The authors declare that the research was conducted in the absence of any commercial or financial relationships that could be construed as a potential conflict of interest.
